# Accelerating Biologics PBPK Modelling with Automated Model Building: A Tutorial

**DOI:** 10.3390/pharmaceutics17050604

**Published:** 2025-05-02

**Authors:** Abdallah Derbalah, Tariq Abdulla, Mailys De Sousa Mendes, Qier Wu, Felix Stader, Masoud Jamei, Iain Gardner, Armin Sepp

**Affiliations:** Certara Predictive Technologies Division, Certara UK Ltd., Level 2-Acero, 1 Concourse Way, Sheffield S1 2BJ, UKfelix.stader@certara.com (F.S.);

**Keywords:** PBPK, biologics, mAbs, oligonucleotides, automation, Simcyp Designer, graphical modelling language, reproducibility, large molecule drug development

## Abstract

Physiologically based pharmacokinetic (PBPK) modelling for biologics, such as monoclonal antibodies and therapeutic proteins, involves capturing complex processes, including target-mediated drug disposition (TMDD), FcRn-mediated recycling, and tissue-specific distribution. The Simcyp Designer Biologics PBPK Platform Model offers an intuitive and efficient platform for constructing mechanistic PBPK models with pre-defined templates and automated model assembly, reducing manual input and improving reproducibility. This tutorial provides a step-by-step guide to using the platform, highlighting features such as cross-species scaling, population variability simulations, and flexibility for model customization. Practical case studies demonstrate the platform’s capability to streamline workflows, enabling rapid, mechanistic model development to address key questions in biologics drug development. By automating critical processes, this tool enhances decision-making in translational research, optimizing the modelling and simulation of large molecules across discovery and clinical stages.

## 1. Introduction

Biologics represent a rapidly growing and diverse class of therapeutic agents that has transformed modern medicine by offering targeted treatment options for complex diseases such as cancer, autoimmune disorders, and rare genetic conditions [1]. Unlike small molecules, biologics are typically large, polar, and structurally complex, which fundamentally shape their pharmacokinetic (PK) and pharmacodynamic (PD) behaviour. The distribution of biologic therapeutics is often permeability-limited, driven mainly by convective transport rather than passive diffusion, while their elimination pathways frequently involve proteolytic degradation, receptor-mediated clearance, or target-mediated drug disposition (TMDD) [2]. These characteristics make biologics PK fundamentally different from that of small molecules, requiring specialized modelling approaches.

Modelling and simulation have become indispensable tools in the drug development process, providing insights that guide decision-making across all stages, from discovery to clinical trials [3]. These approaches reduce the time and cost of drug development by enabling the exploration of different scenarios, optimizing study designs, and predicting clinical outcomes [4]. Physiologically based pharmacokinetic (PBPK) modelling, in particular, stands out as a powerful technique that integrates physiological, biological, and drug-specific information to simulate the absorption, distribution, metabolism, and excretion (ADME) of therapeutic agents in virtual populations [5]. Regulatory agencies, including the U.S. Food and Drug Administration (FDA) and the European Medicines Agency (EMA), have increasingly recognized PBPK modelling as a valuable tool in drug development, supporting dosing strategies, drug-drug interaction assessments, and extrapolations across populations [6].

For biologics, PBPK modelling is indispensable due to their inherently complex distribution and clearance mechanisms. This complexity necessitates a more nuanced modelling strategy to accurately predict the PK of biologics across different patient populations and species [7]. PBPK models for biologics can provide crucial insights into factors such as tissue-specific delivery and the impact of physiological barriers on the level of target engagement, which are essential for effective therapeutic design and optimization.

Despite their advantages, whole-body models for biologics are notably complex, often comprising hundreds of ordinary differential equations (ODEs), thousands of parameters, reactions, and algebraic relationships. This complexity renders manual coding error-prone and, in many cases, impractical. Nonetheless, the inherent modular and systematic nature of PBPK models positions them ideally for automated model building. Automated building leverages predefined building blocks that are automatically assembled, thereby reducing manual coding errors, enhancing reproducibility, and enabling rapid customization and scalability across various biologics PBPK scenarios [8].

This tutorial introduces a biologics PBPK platform built using Simcyp Designer [9]. The platform leverages a modular and adaptable framework that can be efficiently tailored to a variety of biologic modalities. In the following sections, we introduce the fundamental principles of biologics PBPK modelling and demonstrate the power of automated model building in streamlining the construction of biologics PBPK models through case studies covering diverse biologics modalities.

## 2. Whole-Body PBPK Model Structure

PBPK models represent the body as a series of interconnected compartments with each compartment corresponding to an individual organ or tissue (for the purposes of this tutorial, the term ‘organ’ will be used to refer to both organs and tissues). For small molecules, compartments typically follow either perfusion-limited kinetics (common for small, lipophilic drugs), or permeability-limited kinetics (typical for larger, polar molecules). In a perfusion-limited compartment, rapid equilibration between tissue and blood is assumed based on tissue-to-plasma partition coefficients ($K_{p}$), with the time to steady state primarily determined by organ volume and blood flow. In contrast, permeability-limited models subdivide organs into intracellular and extracellular compartments separated by a membrane permeability barrier, making the rate of achieving steady state dependent on the drug permeability across that barrier [10].

In contrast to the relatively simple structure used for small molecules, the complexity of processes underlying the PK of biologics necessitates additional modelling considerations. PBPK models for biologics are typically structured at two levels: whole-body module and organ-level module [11]. While the whole-body module shares similarities with small-molecule models, significant modifications are required to the organ-level modules to capture the unique PK behaviour of biologics. A key distinction is the incorporation of lymphatic flow as an additional transport pathway. Unlike small molecules that predominantly cross capillary membranes via transcellular and paracellular diffusion, large molecules encounter significant barriers to transcapillary transport. Their extravasation from blood into tissues is largely driven by convection and diffusion through paracellular pores. Once in the interstitial space, they rely on lymphatic drainage to return to the systemic circulation [12].

**Figure 1:**
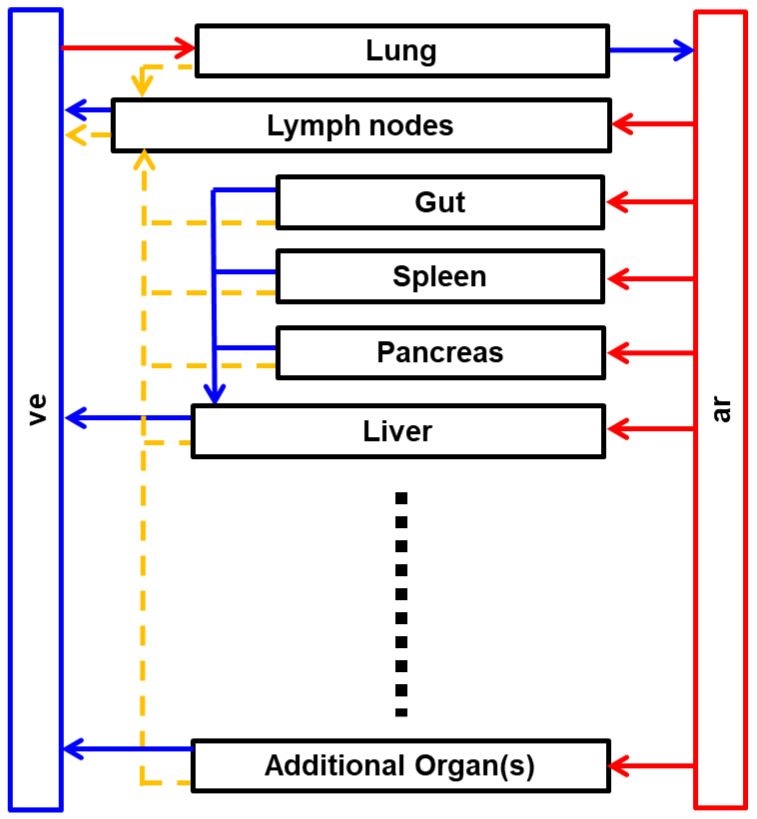
Schematic representation of the whole-body PBPK module of a large molecule. Red arrows represent arterial blood flow, blue arrows represent venous blood flow, and yellow arrows represent lymph flow. ar: arterial blood, ve: venous blood.

In the literature, minimal PBPK models simplify organ representation by lumping all tissues into a single compartment or grouping them into a few compartments based on shared characteristics [13]. In contrast, this tutorial focuses on constructing a whole-body PBPK model, where individual tissues are explicitly represented to capture relevant physiological and PK processes (Figure 1).

**Figure 2:**
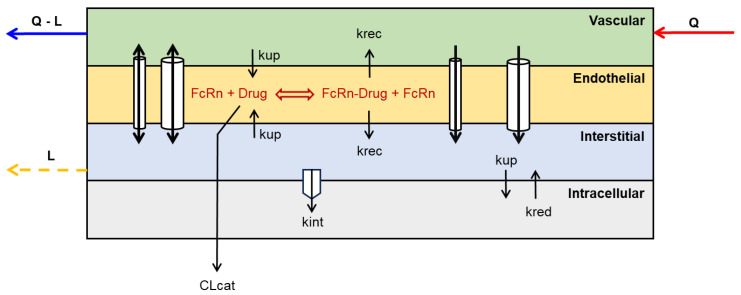
Schematic representation of a typical organ-level PBPK module for large molecules. Small and large cylinders represent the endothelial large and small pores, double-headed arrows crossing cylinders represent diffusion, while single headed arrows represent convection. Q: blood inflow, Q-L; blood outflow, L; lymph flow, kup; fluid phase endocytosis, krec; recycling, kred; redistribution, kint: receptor-mediated endocytosis, and CLcat; catabolic clearance.

The structure of a typical organ-level module is illustrated in Figure 2. The tissue is split into (1) the vascular space (the capillaries within an organ), where biologics are transported into the organ via the arterial blood flow (Q) and out through the venous outflow which is calculated as the difference (Q−L) between blood flow and lymphatic flow (L); (2) the interstitial space. (3) the endothelial endosomal space, which acts as key site for catabolic degradation of large molecules after uptake through fluid-phase endocytosis. Importantly, Fc-containing large molecules, such as IgG-based monoclonal antibodies (mAbs), and albumin bind the neonatal Fc receptor (FcRn) which protects them from catabolic degradation, recycles them back into the circulation or across the tissue, thereby extending their half-life, and (4) the intracellular space is included to represent the organ’s parenchymal cells. In addition to fluid phase endocytosis, those cells can uptake large molecule through receptor mediated endocytosis where internalisation happens as consequence of binding to a cell-surface receptor.

## 3. Mechanistic Determinants of Biologics Disposition

This section explores the key physiological processes governing biologics distribution and clearance, including transcapillary transport, FcRn recycling, renal elimination, and target-mediated drug disposition. Each mechanism is described in the context of PBPK modelling, with relevant equations and parameter considerations provided to support model implementation.

### 3.1. Two-Pore Formalism for Transcapillary Transport

The two-pore model, originally introduced in the 1950s [14], later applied to PBPK models [15] describes the transcapillary transport of large molecule solutes by postulating the existence of two distinct pore populations in the endothelial membrane: small pores and large pores. This model accounts for the extravasation of large molecules via both convection and diffusion as described by Equation (1) and Equation (2), representing transport through both small and large pores, respectively.

(1) $${CL}_{S\_org}=L_{S\_org}\times \left(1-\sigma_{S\_org}\right)+\frac{{PS}_{S\_org}\times {Pe}_{S\_org}}{e^{{Pe}_{S\_org}}-1}\times \left(1-\frac{C_{is\_org}}{C_{v\_org}}\right)$$

(2) $${CL}_{L\_org}=L_{L\_org}\times \left(1-\sigma_{L\_org}\right)+\frac{{PS}_{L\_org}\times {Pe}_{L\_org}}{e^{{Pe}_{L\_org}}-1}\times \left(1-\frac{C_{is\_org}}{C_{v\_org}}\right)$$

where CL is the transcapillary clearance, L is the lymph flow rate, σ is the vascular reflection coefficient, PS is the permeability surface area product, Pe is Peclet number, and $C_{is\_org}$ and $C_{v\_org}$ are the solute concentration in the organ’s interstitial and vascular sub-compartments, respectively. The subscripts *_s_* and *_L_* indicate small and large pore respectively, while *_org_* indicates organ-specific parameters/variables.

The first term in both Equations (1) and (2) represent the convective clearance, which is driven by hydrostatic pressure gradients while the second term describes the diffusive clearance, governed by concentration gradients. The vascular reflection coefficient (σ) determines the extent to which a solute is hindered from crossing the endothelial pores, whereas the Peclet number (Pe) quantifies the balance between convection and diffusion for a given pore type for a given solute. These parameters are typically predicted based on molecular size and organ-specific endothelial membrane porosity characteristics, which are generally assumed to be conserved across animal species and have been estimated using data from a range of large molecule modalities [16]. A detailed description of these calculations can be found elsewhere [17]. Note that many of these parameters are dependent on the solute’s MW. Protein binding in plasma and in the interstitial space can therefore have a significant impact on tissue distribution and calculations are performed for both the bound and unbound biologics to get a reliable prediction of net extravasation of the large molecule.

### 3.2. Catabolic Clearance and FcRn-Mediated Recylcing and Transcytosis

Following uptake into endothelial cells, large molecules enter the endosomal compartments where their fate is determined by the competing processes of lysosomal catabolic degradation and FcRn-mediated recycling. As the endosome undergoes acidification, Fc-containing molecules (and albumin) can bind to FcRn in a pH-dependent non-competitive manner [18]. This interaction protects the bound fraction from catabolic degradation, whereas the unbound molecules are directed towards proteolysis. The FcRn-bound fraction is then transported back to the cell surface on both sides of the endothelial cell layer and released upon exposure to physiological pH, either at the apical (luminal) or basolateral (interstitial) membrane. This process contributes to transcytosis, enabling the movement of Fc-containing molecules across the endothelial barrier [19].

In PBPK models, FcRn interaction can be described using either an equilibrium binding or dynamic binding approach. The Simcyp Designer’s biologics platform employs a dynamic binding model which explicitly accounts for the kinetic rates of FcRn association and dissociation as shown in Equations (3) and (4).

(3) $$\frac{dC_{en_{org}}}{dt}={Kup}_{va_{org}}\times C_{va_{org}}+{Kup}_{is_{org}}\times C_{is_{org}}-\left(\frac{CLcat}{V_{en_{org}}}\times C_{en_{org}}\right)\;\;\;\;\;\;\;-{kon}_{FcRn}\times C_{en\_org}\times {FcRn}_{en\_org}+{koff}_{FcRn}\times {C\_FcRn}_{en\_org}$$

(4) $$\frac{d{C_{FcRn}}_{en_{org}}}{dt}={kon}_{FcRn}\times C_{en_{org}}\times {FcRn}_{en_{org}}-{koff}_{FcRn}\times {C_{FcRn}}_{en_{org}}\;-krec\times {C\_FcRn}_{en\_org}\;\;\;\;\;\;$$

where Kup is the endocytosis uptake rate constant, C is the free concentration of the drug, CLcat is the catabolic clearance, V is the organ’s sub-compartment volume, ${kon}_{FcRn}$ and ${koff}_{FcRn}$ are the FcRn association and dissociation rate constants, FcRn is the concentration of free FcRn, C_FcRn is the concentration of drug-FcRn complex, and krec is the recycling rate constant of the drug-FcRn complex. The subscripts *_en_* and *_va_* indicate the endothelial and vascular sub-compartments, respectively.

### 3.3. Renal Elimination

The kidneys play a crucial role in the clearance of some biologics, primarily through glomerular filtration. The glomerular filtration barrier exhibits both size-selectivity and charge-selectivity, which influence the extent to which large molecules can pass into the filtrate [20].

The size-selectivity of the glomerular barrier can be effectively described by the two-pore model, which establishes a relationship between molecular size (or MW) and the glomerular sieving coefficient (GSC), the fraction of a solute that is freely filtered relative to water [16]. For molecules larger than ~60 kDa, the GSC approaches zero, indicating negligible filtration, whereas smaller molecules are more readily filtered. However, plasma protein binding, such as binding to albumin, can substantially reduce the fraction available for filtration, further limiting renal clearance. Renal filtration effectively captures the general relationship between protein half-life and molecular weight [21].

Beyond filtration, the kidneys contribute to large molecule elimination via lysosomal degradation, peritubular reabsorption, and intracellular catabolism in proximal tubule cells [22]. The overall net filtration clearance of large molecules from the renal vascular compartment can be described by Equation (5).

(5) $${CL}_{filtration}=GFR\times \theta \times f_{up}\times (1-f_{reab})$$

where ${CL}_{filtration}$ is the net filtration clearance, GFR is the glomerular filtration rate, PR is the partition ratio, θ is the GSC, $f_{up}$ is the fraction unbound in plasma, and $f_{reab}$ is the fraction reabsorbed intact.

### 3.4. Target Mediated Drug Disposition & Receptor Mediated Endocytosis

Target-mediated drug disposition (TMDD) describes a pharmacokinetic phenomenon in which a drug binds with high affinity and selectivity to a pharmacological target, leading to nonlinear clearance [23]. This characteristic is particularly common for biologics such as mAbs and fusion proteins, that have strong and specific target interactions. TMDD has a pronounced impact at low drug concentrations, where target binding is not saturated, resulting in rapid, capacity-limited clearance that exceeds the rate of non-specific clearance. In contrast, at higher drug concentrations, the target becomes saturated, and the contribution of TMDD to drug elimination diminishes, shifting clearance balance toward nonspecific nonsaturable pathways such as macropinocytosis and lysosomal degradation [24].

**Figure 3:**
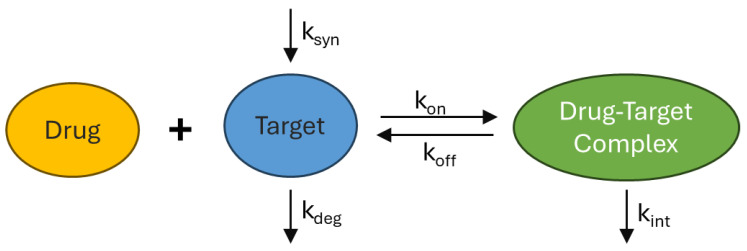
A schematic representation of the full target mediated drug disposition (TMDD) model.

A widely accepted framework for describing TMDD is the full TMDD model, outlined in Figure 3 [25], which explicitly represents the key processes governing drug-target interactions. In this model, the drug binds reversibly to its target, with the association and dissociation dynamics described by rate constants governing the formation and dissociation of the drug-target complex. The target itself is subject to continuous turnover, synthesized at a zero-order rate and degraded via a first-order process. Once formed, the drug-target complex undergoes internalization, a process often modelled as a first-order event leading to intracellular degradation.

For soluble targets, complex internalization is typically assumed to occur at a rate comparable to the free drug’s elimination rate and shares the same macropinocytosis-endosomal trafficking pathway, whereas for membrane-bound targets, internalization is generally modelled at a rate equal to the receptor degradation rate, reflecting the natural turnover of the receptor-ligand complex [26]. The extent to which TMDD affects drug clearance depends on the abundance and turnover of the target, as well as the binding affinity of the drug.

Beyond its role in drug elimination, TMDD can also serve as a mechanism for intracellular drug delivery. When a drug binds to a membrane-bound receptor, receptor-mediated endocytosis (RME) facilitates its transport from the interstitial space into the intracellular compartment [27]. This mechanism is commonly exploited in the design of antibody-drug conjugates (ADCs) and targeted therapeutics aimed at intracellular targets [28]. Since TMDD and RME follow the same fundamental principles, they can be described using a similar mathematical framework. The key distinction lies in the fate of the internalized drug. In TMDD as a clearance mechanism, the internalized drug-target complex undergoes degradation, leading to drug elimination. In RME, the internalized drug is instead released within the cell to exert its pharmacological effect.

## 4. Introduction to Simcyp Designer

Simcyp Designer is a model-building platform designed to facilitate the construction and editing of ODE-based models through a graphical user interface (GUI). The platform features a modular architecture, enabling hierarchical representation of model components and efficient handling of complex biological interactions.

To represent complex ODE models graphically, Simcyp Designer employs three fundamental types of objects: nodes, edges, and containers. Nodes serve as the building blocks of a model and are categorized into quantity nodes and quantity-modifying nodes. Quantity nodes store numerical values and include:

(a) “Species” nodes, which represent dynamic states in the ODE model. Note that the term “Species” here refers to mathematical states, not biological species. When referring to different biological species, we explicitly use “animal species”, and
(b) “Parameter” nodes, which define parameters in the ODE model.

Quantity-modifying nodes modify the values of quantity nodes and include:

(a) “Reaction” nodes, which consume and produce “Species”, i.e., represent ODE terms,
(b) “Assignment” nodes, which assign values to quantity nodes based on evaluated expressions. These can be either repeated assignments for time-varying quantities or initial assignments for fixed quantities.
(c) “Dosing Plan” nodes, which specify times and amounts (or rates) of doses to “Species”.

Edges define relationships between nodes by connecting quantity nodes to quantity-modifying nodes. These connections allow a quantity to be modified by a node, brought into its scope, or both. Finally, containers are graphical objects that contain model components. A “Compartment” is the primary container which represents a generalised volume that contains “Species” and holds a value that maybe assigned using “Assignment” nodes. A more detailed description of these graphical objects and their corresponding model components is provided in Supplementary S1, while a comprehensive explanation of how a model can be defined graphically and translated into simulation-ready code is described in [9]. Readers are strongly encouraged to consult both Supplementary S1 and reference [9] to facilitate a clearer understanding of the modelling framework and to follow the concepts presented throughout the rest of this manuscript.

Beyond basic graphical objects, Simcyp Designer includes modularity features that streamline the construction of complex models. These features facilitate model reusability, allowing specific components to be applied multiple times, and enable the automated generation of redundant reaction schemes. This functionality significantly enhances scalability, making it possible to construct large and intricate models efficiently. The benefits of these modular features are particularly evident in whole-body PBPK modelling of biologics, where different drug types interact with multiple targets, exhibit organ-specific expression patterns, and are governed by distinct biological processes. The following subsections introduce these modularity features with minimal examples to illustrate their implementation, followed by case studies demonstrating their application in building and utilizing the biologics PBPK platform.

### 4.1. Quantity Arrays

**Figure 4:**
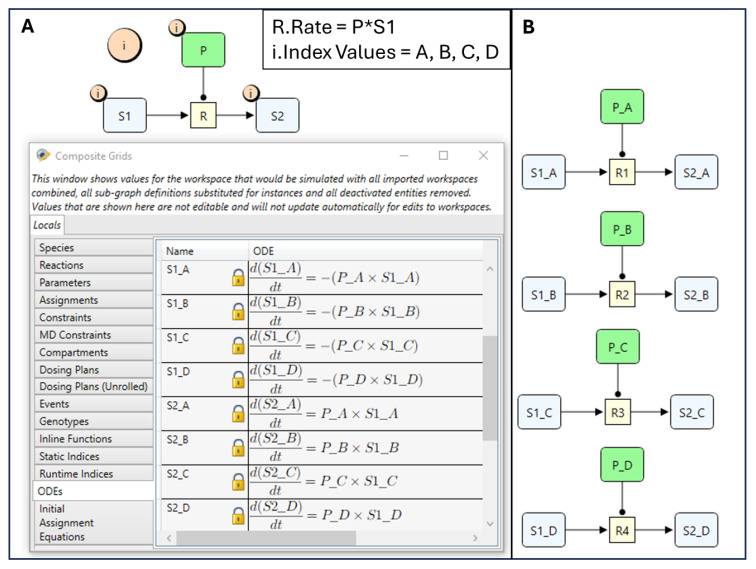
Index expanded reaction scheme (**A**) and an equivalent manually created reaction scheme (**B**). The inset shows the “Rate” property of the reaction “R” and the “Index Values” property of the index “i”. Both reaction schemes produce the same set of ODEs shown in (**A**) with the index expanded scheme much more compact. “*” denotes multiplication.

Arrays of quantities, i.e., species, parameters, or compartments, can be created by attaching an index to the quantity. When an index is attached to a quantity, the number of values the quantity can hold will be multiplied by the range of the index (the number of values it has). For example, if an index “i” with values A, B, C, and D is attached to a parameter “P” then it will create an array with the four components P_A, P_B, P_C, P_D. This can be a very handy tool for creating large number of quantities, reactions, automatically as illustrated in Figure 4 where a manually created reaction scheme is shown together with its index expanded reactions equivalent. Both implementations produce the same set of ODEs (shown in the figure), however, with the index expanded option much easier to construct, communicate, and maintain and will not contain typing mistakes. This is a very commonly encountered scenario in PBPK where the same reaction pattern needs to be repeatedly created in different organs as will be seen in the case study examples.

### 4.2. Reusable Subgraphs

While indices enable the rapid generation of multiple species and reactions, reusable subgraphs allow for the replication of entire model structures. This is implemented through Subgraph Definitions and Subgraph Instances. A Subgraph Definition serves as a blueprint for a submodel, while Instances are its replicated versions within the main model. Objects marked as Exposed within the Subgraph Definition can be interacted with externally, appearing as channels on each Instance. Any modifications made to the Subgraph Definition are automatically propagated across all linked Instances, ensuring consistency.

**Figure 5:**
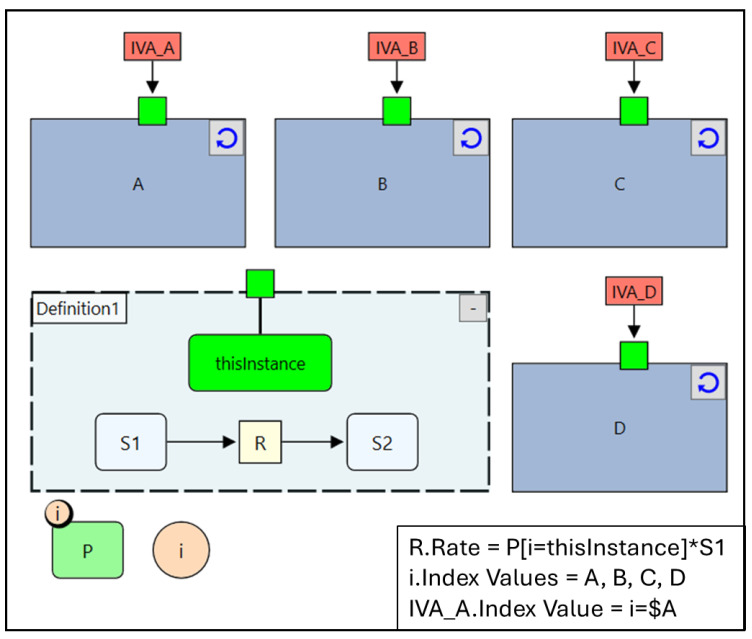
Reusable subgraphs used to construct the same ODE system in Figure 4. The inset shows the rate expression for the Reaction “R”, Index Values for the Index “i”, and Index Value for the Index Value Assignment “IVA_A”. “*” denotes multiplication, circular arrows at the top right corner of an instance represent button that resync the instance with the definition, small green squares at the edge of an instance represent the corresponding exposed node (Index Value Node) in the corresponding definition.

To illustrate the functionality of reusable subgraphs, we reconstruct the ODE system from Figure 4 using this approach. First, we define a subgraph containing the reusable reaction structure: a reaction R that consumes S1 and produces S2, as shown in Figure 5. Next, four Instances of this subgraph are created to represent reactions R1 to R4. Since each reaction requires a different parameter value “P”, an Index Value node, labelled “thisInstance”, is introduced in the subgraph definition (represented by the green rectangle in Figure 5). This node is used to select the correct element of the parameter array “P”, such that each instance (e.g., Instance A, B, C, etc.) uses its corresponding value (P_A, P_B, etc.).

This selection is achieved by incorporating an index specifier into the reaction rate expression: “P[i=thisInstance]∗S1”. Here, the value of “P” used in the reaction rate depends on the value stored in “thisInstance”. To assign different values to “thisInstance” for different instances, we mark it as an Exposed node. Exposed nodes are visually distinguished by connections to a channel at the boundary of the Subgraph Definition and corresponding channels at the Instance level. Finally, an Index Value Assignment (IVA) node is used to set “thisInstance” for each subgraph instance. For example, in Instance A, the assignment expression “i=$A” ensures that “thisInstance” holds the value A, selecting P_A for that instance. The same approach is applied to the remaining instances, ultimately reconstructing the original ODE system from Figure 4.

We note that subgraph instances are designed to read, but not modify, global parameters, i.e., parameters that are created outside of the subgraph definition and don’t have any modifier edge scoping them to a specific quantity modifier. This ensures that global parameters remain consistent throughout the model and prevents conflicts or redundancies that could arise from multiple subgraph instances attempting to overwrite shared values. The Index Value Assignment nodes are used to direct subgraph instances to specific elements within global parameter arrays. These nodes allow each instance to access only the relevant array component, ensuring that the global parameter itself is not altered. For example, in a parameter array representing tissue-specific FcRn concentrations, different subgraph instances representing various tissues can each access the appropriate element of the array via its assigned index value, without any possibility of cross-interference. Because of this strict separation between parameter access and parameter definition, the global parameter remains consistent throughout the model, preventing any unintended modifications or conflicts.

While indices provided a more compact and straightforward approach in the previous example, reusable subgraphs become significantly more efficient when replicating complex model structures. As demonstrated in the first case study, subgraph instances were used to replicate the organ-level module within a whole-body PBPK model. This approach enables the efficient generation of multiple organ compartments as copies of the organ-level module while allowing each organ to utilize its own distinct, organ-specific parameter values.

### 4.3. Quantity Lists

So far, we have demonstrated how indices and reusable subgraphs can be used to automatically generate a series of reactions. In these examples, the number of substrate and product species was predefined. However, in constructing a biologics PBPK platform, it is often necessary to define reactions for an unknown number of species. For instance, the two-pore extravasation reaction applies to all soluble species, but the number of soluble species varies across different modelling scenarios (e.g., a mAb with a soluble vs. membrane-bound target). To avoid redefining the two-pore reaction each time a new soluble species is introduced, a more flexible approach is required. This is where quantity lists become essential.

**Figure 6:**
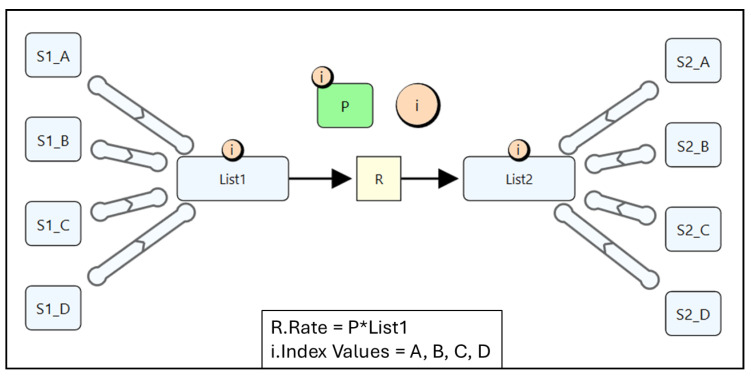
Species Lists used to construct the same ODE system in Figure 4. The inset shows the rate expression for the Reaction “R”, Index Values for the Index “i”, “*” denotes multiplication.

To illustrate their utility, we revisit the same example used for indices and reusable subgraphs. In Figure 6, we demonstrate how to construct a reaction scheme that produces the same ODE model shown in Figure 4, but now using quantity lists. In this setup, individual species are first created and then grouped into a Species List. This is achieved by connecting them with a “Quantity List Meta Edge”, which indicates that a species is an element of the connected list. The meta edge is visually represented as a thick edge with rounded circular ends and an arrow in the middle, signifying the direction of membership (pointing towards the containing list).

When a Species List is connected to a reaction, either as a substrate or a product, all species within the list automatically inherit that reaction. Lists can be easily expanded by adding new elements, which will also inherit any associated reactions. This makes quantity lists a highly flexible tool for encoding reactions that involve an undefined number of species, allowing for easy and quick model adaptation without manual reconfiguration. Note that Several verification checks are enforced by Simcyp Designer to ensure consistency between list indexing and quantity elements and to maintain the correct reaction scheme. These checks must pass before simulation or code generation can proceed. The main verification checks are outlined in Supplementary S2.

Simcyp Designer allows for a single quantity to belong to more than one quantity list. This flexibility supports model reuse and modular construction. However, Simcyp Designer does not automatically detect or resolve overlapping contributions to a species’ ODE when it appears in multiple quantity lists that participate in distinct reactions. As such, it is the modeler’s responsibility to ensure that the cumulative contributions to each species’ equation are consistent with the intended biology and do not lead to double-counting or overcounting of mass flow. To mitigate this risk, best practices include ensuring that distinct reactions are non-overlapping.

### 4.4. Virtual Population (VPop) Files

Simcyp Designer enables the execution of simulations with different initializations of some or all model quantities through VPop files, which are provided in CSV format. A VPop file consists of an “ID” column, which assigns a unique integer identifier to each distinct set of quantity values, an optional “LABEL” column for descriptive annotations, and one or more additional columns corresponding to the quantities being overridden. Each row in the file represents a complete set of quantity values to be used in a single simulation.

When a model is simulated using a VPop file, multiple simulations are performed, one for each row (excluding the header). In each simulation, the corresponding values from that row replace the default model quantities, allowing for efficient exploration of different parameter sets. Table 1 provides an example VPop file that can be used to simulate the model shown in Figure 6. Notably, a VPop file does not need to include all quantities in the model; only the quantities intended for modification are to be specified.

VPop simulations have several applications. They can be used for population simulations, where each row represents an individual with unique physiological parameters. This can be a very handy tool for predicting drug PK under physiological extremes, e.g., renal/hepatic impairment or paediatric populations. Additionally, as demonstrated in Case Study 1, VPop files can facilitate animal species-specific simulations, enabling the same model structure to be applied to different animal species. In this case, each row of the VPop file represents the physiological parameter set for the respective animal species. They can also be employed to evaluate different dosing scenarios by systematically varying drug administration parameters.

Inter-parameter dependencies or physiological constraints (e.g., ensuring that organ volumes add up to total body volume) are not automatically enforced by Simcyp Designer. However, users can encode such dependencies explicitly within the model workspace using assignment nodes. Parameters defined through assignment nodes are computed during simulation initialization and therefore cannot be overridden through the VPop file. This mechanism provides a flexible and robust way to enforce constraints or derive dependent parameters from user-defined inputs. For instance, a user may have an organ called “Other” to represent unmodelled organs and assign its volume as the difference between total body volume and the sum of individual organ volumes provided in the VPop file. This guarantees that all organ volumes add up correctly, maintaining physiological plausibility across simulations. This design allows users to maintain control over parameter relationships while still benefiting from the flexibility of row-wise parameter variation provided by the VPop file.

## 5. Application Case Studies

### 5.1. Case Study 1: A Model for Monoclonal Antibodies

The biologics market is largely driven by mAbs, which represent the biggest and most extensively developed class of biologic therapeutics [29]. Understanding their whole-body PK and target engagement requirements in relevant organs during early drug discovery is crucial for optimizing therapeutic strategies. PBPK modelling provides a robust framework to investigate mAb PK across different tissues and animal species, enabling informed decision-making [30].

Despite some variability, mAbs exhibit relatively consistent PK behaviour, both among themselves and in comparison with other large molecules [31,32]. This consistency makes them well-suited for a modelling platform that captures shared attributes while allowing for minimal modifications to tailor the model to specific agents. Therefore, the biologics platform has been developed using a mAb example as a foundational model.

In this case study, we demonstrate how the mAb model was developed by leveraging key features of Simcyp Designer. The subsequent case studies will illustrate how this model can be rapidly adapted to accommodate different biologics modalities and modelling scenarios with minimal adjustments, eliminating the need to rebuild models from scratch while maintaining accuracy and efficiency.

1. Indices

The platform model is structured around several key indices to efficiently manage organ-specific and biochemical interactions. The primary index, the “organ” index, encompasses 21 values corresponding to different organs in the whole-body model, along with two additional values representing the arterial and venous compartments. This indexing system enables organ-specific parameters to be stored in a 23-dimensional array, facilitating defining organ-specific parameters.

In addition to the organ index, the platform model includes the “soluble” index, which is attached to the “Soluble” species lists. As will be detailed later, those lists have been created to simplify the definition of reactions shared among soluble species, e.g., two-pore reaction. While the current model only incorporates the mAb as a soluble species, this index provides flexibility for the future inclusion of other soluble components, such as a soluble target. Similarly, the “circulating” index is incorporated to enable circulation reactions to be defined for any number of Species in circulation (blood or lymphatic). Finally, the model includes the “doseTarget” index, which distinguishes between vascular species (for intravenous dosing) and interstitial species (for extravascular routes such as subcutaneous or intramuscular administration). This allows both dose amount and route of administration to be defined using VPop files. How these indices are applied will become clearer as further components of the platform model are described.

2. Parameters Module

**Figure 7:**
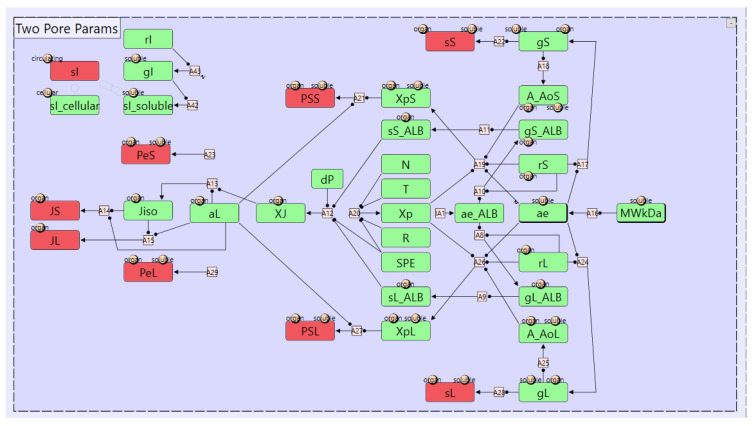
The module representing the definition of the two pore parameters within the mAb PBPK model. The input parameter to this module is the soluble species molecular weight “MWkDa”. The output parameters are shown in Red, while the intermediate parameters are shown in green. Black lines represent the parameter dependencies while pink squares represent assignment nodes. sS, and sL are the vascular reflection coefficient for the small and large pores, PSS and PSL are the permeability surface area products for the small and large pores, PeS and PeL and the Peclet’s numbers for the small and large pores, JS and JL are the lymph flow rates through the small and large pores, respectively, and sl is the lymphatic vessels reflection coefficient.

The “Parameters” module encompasses the definition of model parameters. A key sub-module within the “Parameters” module is the “Two-Pore Params” module, illustrated in Figure 7. This module implements the calculation of two-pore parameters as described in [16]. The input to this module is the soluble species MW parameter “MWkDa”. This parameter has the “soluble” index attached to it which means a value is required for each soluble species in the model. That index is also attached to some output parameters meaning they will be automatically calculated and output for each soluble species in the model, eliminating the need for defining those complex calculation for each soluble species separately. Similarly, organ-specific parameters such as the small pore radius “rS” are indexed by the “organ” index. Some parameters depend on both organ-specific and soluble species-specific parameters. For example, the output parameter “sL”, the vascular reflection coefficient for large pores, which has both the “organ” and “soluble” indices attached. Parameters can either have fixed values or be dynamically calculated. Fixed parameters are assigned explicit values using the “Value” property of the parameter object (or through Vpop files). Alternatively, parameters can be computed based on other parameters using assignment nodes as shown in Figure 7.

**Figure 8:**
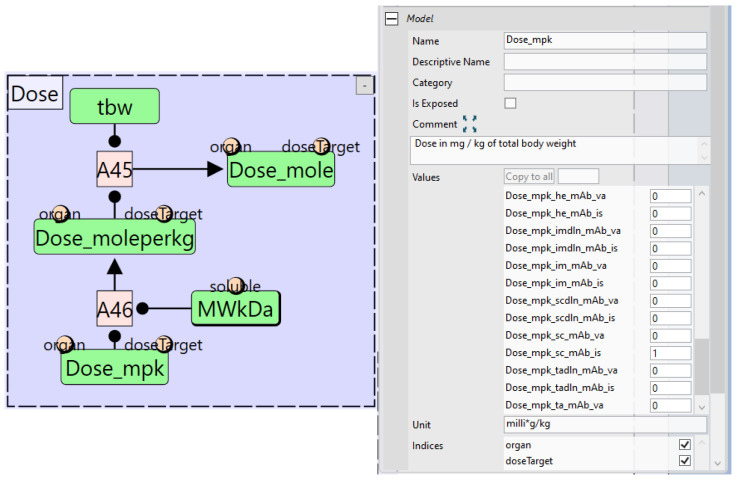
The dose calculation module (**left**) and the properties panel of the input parameter “Dose_mpk” (**right**). “Dose_mpk” is the dose in mg/kg units. The properties panel show that a dose is defined for the “Dose_mpk_sc_mAb_is” element of the array indicating the dose is for the subcutaneous “sc” organ targeting the “mAb_is” species. tbw is the total body weight, and MWkDa is the molecular weight of the biologic.

To facilitate inputting dosing information, the “Parameters” module also incorporates a module for “Dose” calculation shown in Figure 8. The input to this module is the “Dose_mpk” parameter which has the dose value in mg/kg. This parameter has both the “organ” and the “doseTarget” indices attached to it, meaning that a value needs to be defined for each unique combination of elements of both indices. For example, an SC dose of 1 mg/kg would require the use of “organ” index value of “sc” for subcutaneous tissue and “doseTarget” index value of “mAb_is” for the mAb in the interstitial compartment. Therefore, the value for “Dose_mpk_sc_mAb_is” should be set to 1 while all other values set to 0. This is then converted to total dose in moles using the molecular weight “MWkDa” and total body weight “tbw” parameters.

3. Organ-level subgraph definition

**Figure 9:**
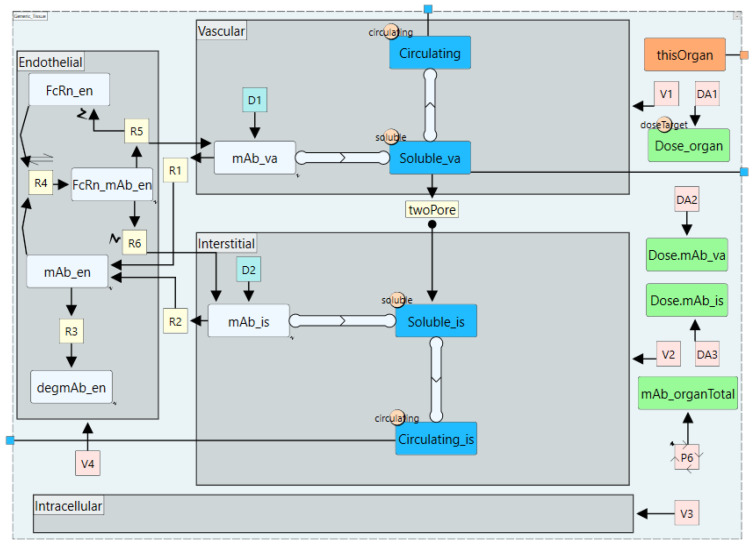
The organ-level module defined in a subgraph definition. Blue nodes are species lists, light blue nodes are species, yellow nodes are reactions, orange node represents an index value node, green nodes are parameters, pink nodes are assignment nodes, and grey rectangle are compartments. Thick light blue edges represent species list meta edges and the arrow in the middle represents the direction of list membership. Small rectangles at the edge of the definition represent the definition interface (exposed nodes). The suffix va: vascular, is: interstitial, and en: endothelial.

The subgraph definition “Generic_Tissue” represents the organ-level model (described under section Whole-body PBPK model structure) and includes model components that are common across organs/tissues as shown in Figure 9. The key elements of the “Generic_Tissue” definition are as follows:

- Index value node

The index value node enables different instances of a subgraph definition to use distinct parameter values. In this model, it ensures that organ-specific parameters, such as sub-compartment volumes, are correctly assigned to the respective instance. To achieve this, an index value node called “thisOrgan” is created within the subgraph definition and exposed so that its value can be set at the whole-body level module.

This index value node is then used as an index specifier in reaction rate or assignment node expressions that reference organ-specific parameters. For example, in Figure 9, the “V1” node, which assigns a volume value to the vascular compartment, contains the expression “V_va[organ=thisOrgan].” This instructs the model to retrieve the appropriate value from the vascular volume “V_va” array corresponding to the “Index Value” stored in “thisOrgan.” As a result, when “thisOrgan” is assigned different values at the whole-body level model for different organs, the corresponding organ-specific parameter values are automatically applied, ensuring organ-specific behaviour without the need for repeatedly define parameters for each organ.

b. Compartments

Four sub-compartments are included in the organ-level model: vascular, endothelial, interstitial, and intracellular. Each compartment’s volume is assigned via an assignment node (V1 to V4 in Figure 9) to ensure the use of organ-specific values. While the intracellular compartment does not contain any species in the current model, it is included to enable future expansions where intracellular processes may be relevant and to facilitate the calculation of the whole-tissue drug concentration, “mAb_organTotal”.

c. Dosing Plan

In this model, dosing plans are linked to two species: “mAb_va” and “mAb_is”, representing the monoclonal antibody in the vascular and interstitial compartments, respectively. The dose amount is obtained from the “Dose_mole” parameter array using the assignment node “DA1”, which applies the expression “Dose_mole[organ=thisOrgan]” to extract the organ-specific dose and assign it to the “Dose_organ” parameter. The assignment nodes “DA2” and “DA3” then distribute the dose to the appropriate species, with the expressions “Dose_organ[doseTarget=$mAb_va]” and “Dose_organ[doseTarget=$mAb_is]” directing the dose to the parameters “Dose.mAb_va” and “Dose.mAb_is”, respectively. Finally, “Dose.mAb_va” defines the amount property of the dosing plan “D1”, while “Dose.mAb_is” defines the amount property of the dosing plan “D2”, ensuring that all elements of the “Dose_mole” array are correctly assigned to their respective dosing plans in the full model.

d. Species and Species Lists

Species of the subgraph definition include the mAb species in various compartments, “FcRn_en” in the endothelial compartment, “FcRn_mAb_en” for the mAb–FcRn complex, and “degmAb_en” for endothelial degraded mAb (all species are depicted as light blue rectangles in Figure 9). Each species has its own set of reactions, which will be detailed in the following section. However, some reactions are common to groups of species, such as the blood inflow and outflow in the vascular compartment, or the two-pore-based extravasation that all soluble species in the vascular compartment undergo. To streamline the implementation of these shared reactions, species are grouped into “Species Lists”. As shown in Figure 9, the species lists (highlighted in blue) include “Soluble_va”, which has a “quantity list meta edge” connecting the “mAb_va” species to it. The edge has an arrow pointing toward the list signifies membership, thereby enabling “mAb_va” to automatically inherit the “twoPore” reaction. Similarly, adding any new soluble species, such as a soluble target, only requires linking them to the “Soluble_va” list to ensure they inherit the same reaction. Additionally, since the “Soluble_va” list is a member of the “Circulating_va” list, its species also inherit the reactions defined for “Circulating_va”. Although the “Circulating_va” list does not have reactions defined within its subgraph, it is marked as exposed, meaning it possesses proxy channels that interact with reactions external to the definition. Consequently, “mAb_va” and all species within the “Soluble_va” list automatically inherit the blood inflow and outflow reactions that will be specified later at the instance interface.

e. Reactions

**Figure 10:**
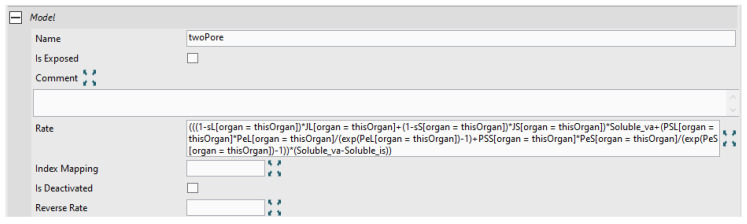
Reaction properties for the two-pore reaction showing the rate expression. Organ specific parameters are specified with the index specifier [organ=thisOrgan]. “Soluble_va” and “Soluble_is” are proxies (Species Lists) for the soluble species in the vascular and interstitial compartments, respectively. “sL”, “JL”, “sS”, “JS”, “PSL”, “PeL”, “PSS”, “PeS” are the two pore parameters calculated for each unique organ/soluble species combination as in the two pore parameters module. “*” denotes multiplication.

Reactions represent terms in the ODE equations of the model and are represented by yellow squares in Figure 9. These include “R1” and “R2”, which are the uptake reaction from vascular and interstitial space into the endothelial space, “R3” which represents catabolic degradation of “mAb_en”, “R4” representing reversible binding to FcRn, and “R5” and “R6” representing the recycling of the bound mAb back to the vascular and interstitial spaces, respectively. As with assignment nodes, reactions can use organ-specific parameters through the index specifiers referencing the index value node “[organ=thisOrgan]”. The “twoPore” reaction is has a rate expression that represents Equations (1) and (2) and uses the “Soluble_va” and “Soluble_is” as proxies for members of both lists as shown in Figure 10.

4. Whole-body level module

**Figure 11:**
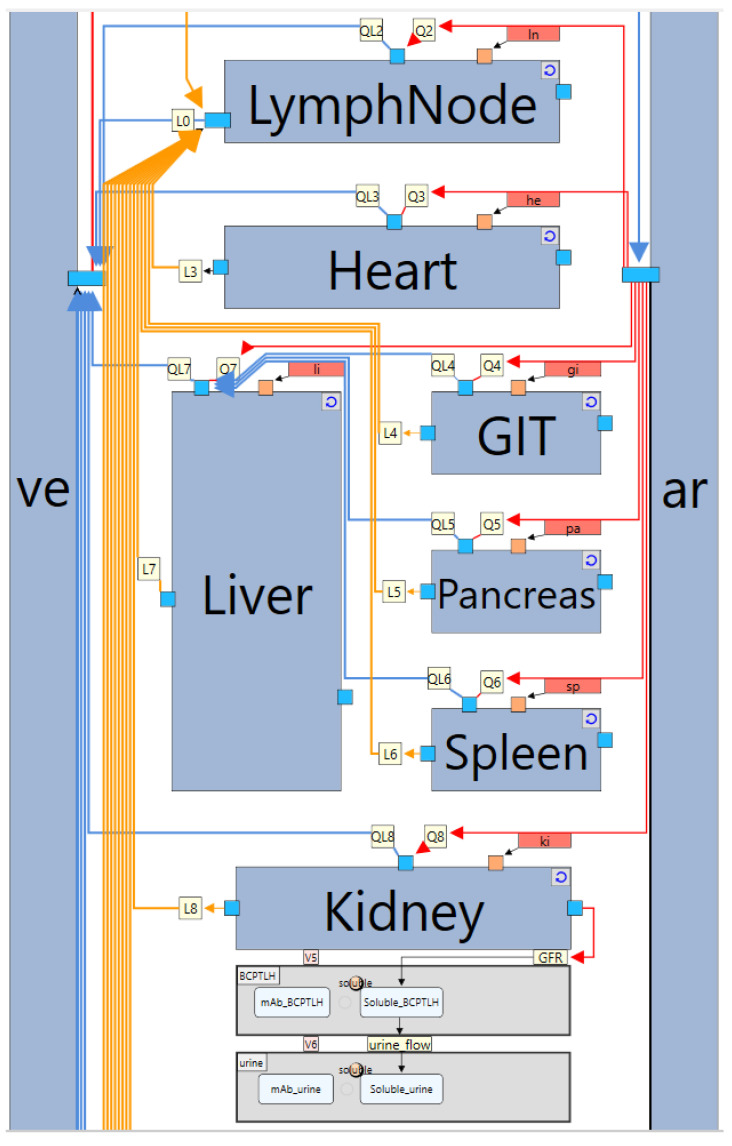
A representative section of the whole-body level module. Large steel blue rectangles represent instances (copies) of the “Generic_Tissue” definition. Channels (small squares) on instances represent exposed nodes from the linked subgraph definition. Red arrows/lines represent arterial blood flow, blue arrows/lines represent venous blood flow, yellow arrows/lines represent lymph flow, red rectangles represent index value assignment nodes, yellow squares represent reactions (Q for arterial flow, QL for venous flow, L for lymphatic flow, GFR for glomerular filtration), and grey rectangles represent compartments. ve: venous blood, ar: arterial blood, BCPTLH: lumped compartment of the Bowman’s capsule, proximal tubules, and loop of Henle.

The whole-body level model is constructed by creating multiple instances of the “Generic_Tissue” subgraph definition, with each instance representing a distinct organ. These instances are then interconnected through reactions that represent the physiological blood and lymph flow, ultimately forming the complete whole-body model. A representative section of this model is illustrated in Figure 11. As shown, each instance contains channels, which correspond to exposed nodes from the subgraph definition. These exposed nodes can interact with components of the model that are external to the instance. For example, blood flow reactions (whose names start with “Q” for arterial flow or “QL” for venous flow) are linked to channels representing the “Circulating_va” species list, while lymph flow reactions (names start with “L”) are linked to channels representing the “Circulating_is” species list. The specific node to which each channel corresponds can be identified through the “Reference Node” property of the channel, ensuring proper mapping of flow reactions across different organs.

Index value assignment nodes (shown as red rectangles in Figure 11) ensure that each organ has its unique index value and therefore can use its correct organ-specific parameter values. For example, the index value assignment of the heart has the Index Value property set to “organ=$he”.

The design of the whole-body module ensures maximum efficiency in model construction. It includes only the components that remain consistent across different biologics modalities, animal species, and most modelling scenarios. As a result, the whole-body model needs to be built only once and can be applied to any biologic without requiring reconstruction or modification, saving hours of tedious, error-prone, unnecessary work. The organ-level instances automatically update in response to any changes made to the linked subgraph definition, ensuring seamless adaptability.

Despite its standardized structure, the whole-body model remains highly flexible. Users can customize specific organs or incorporate additional organs when needed. The flexibility of incorporating additional organs is demonstrated in the IgG pregnancy case study discussed later, where new instances are added to represent the placenta and foetus. Similarly, Figure 11 illustrates an organ-specific customization for the kidney, which has distinct physiological characteristics as an eliminating organ. To account for glomerular filtration, a reaction labelled “GFR” was incorporated, enabling transport of “Soluble_va” species list elements from the kidney’s vascular compartment into a lumped compartment representing the Bowman’s capsule, proximal tubules, and loop of Henle (BCPTLH). Additionally, a urine compartment was introduced, with the “urine_flow” reaction linking it to BCPTLH. These modifications highlight the model’s flexibility, demonstrating that it can be readily extended and tailored to accommodate diverse physiological and pharmacokinetic scenarios.

While building such a comprehensive model from scratch may seem daunting, it only needs to be constructed once. Once established, the framework can be seamlessly adapted to nearly any biologics PBPK modelling scenario without the need for extensive reconstruction. This adaptability significantly enhances efficiency and reduces the risk of errors associated with manual reimplementation [8]. In the following two case studies, we demonstrate this flexibility: first, by extending the mAb platform model to develop an IgG PBPK model in pregnancy, and second, by adapting the same framework to describe the pharmacokinetics of targeted oligonucleotides.

5. Cross-species simulations

**Figure 12:**
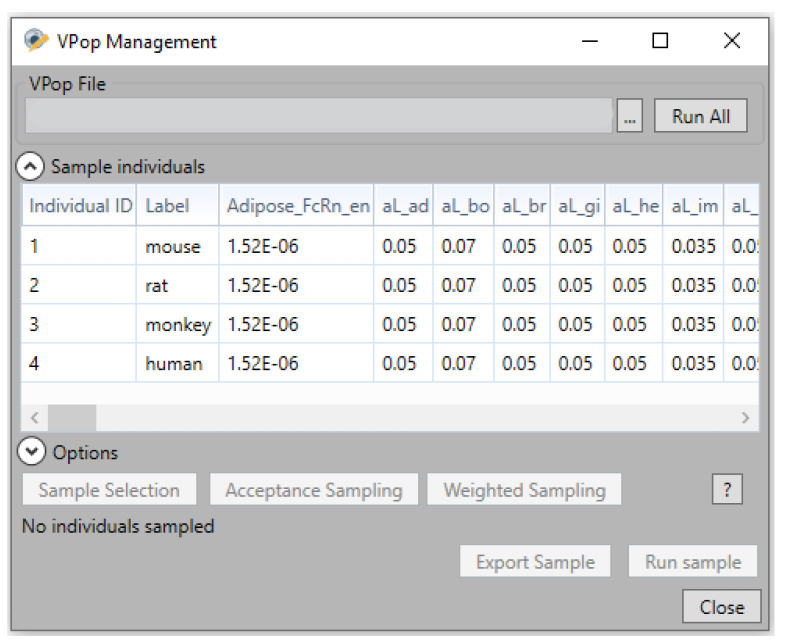
VPop management window in Simcyp Designer.

Since model structure is common across different animal species, it can be used to simulate those animal species by accounting for the difference in physiological parameter values which can be easily done through VPop files. In this case, each row in the file is used to represent different animal species while each column represents a physiological parameter that varies across different animal species, e.g., tissue volumes, flow rates, FcRn abundance, etc. See Figure 12 illustrating an example of a Vpop file containing physiological parameter values for four different species, mice, rats, monkeys and humans. Additionally, VPop files could be used to simulate different scenarios. For example, different doses, dosing regimens or routes, or different target abundances or binding affinities. Basically, any scenario that can be represented as set of parameter values can be simulated as VPop row.

### 5.2. Case Study 2: Pregnancy IgG Model

Clinical trials, involving pregnant patients, are often necessary for new drug developments, but ethical constraints and recruitment challenges present significant barriers. PBPK models have been instrumental in predicting small molecule drug PK during pregnancy, informing study design and dose adjustments [33]. However, PBPK models for mAbs in this population remain underdeveloped [34].

Pregnancy leads to reduced IgG concentrations in otherwise healthy women, even in the absence of infection or autoimmune disease. This decline is primarily attributed to increased plasma volume (haemodilution), active IgG transport to the foetus, and immunoregulatory changes [34]. Foetal IgG originates primarily from the maternal transfer via the placenta, only approximately 0.2% of total IgG in cord blood is of foetal origin [35]. Transfer is minimal during the first trimester but increases significantly in the second and third trimester, reaching adult levels by term [34]. This process, involving endocytosis and transcytosis via the FcRn, facilitates the placental transfer of both endogenous IgG and therapeutic mAbs. Therefore, understanding IgG transfer across the placenta can be of paramount importance for predicting foetal exposure to maternally administered mAbs.

**Figure 13:**
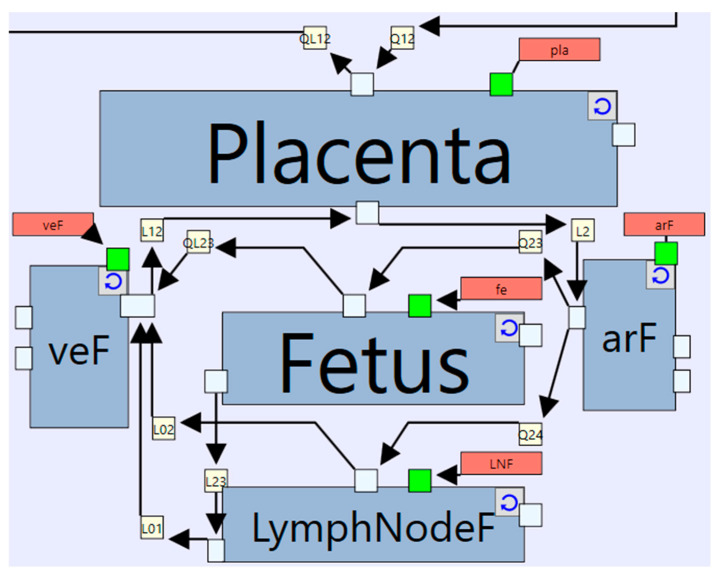
Feto-placental structure added to the whole-body level model. arF: fetal arterial blood, veF: fetal venous blood, Fetus: is the lumped organ representing the whole fetal body, LymphNodeF: is the fetal lymph nodes.

An IgG model in pregnancy can be easily built by adapting the platform mAb model. This can be achieved by creating additional instances of the “Generic_Tissue” definition to represent the placenta and the foetal body as shown in Figure 13. The “Placenta” instance represents the maternal vascular compartment, a shared endothelial compartment of the mother and foetus, and the foetal vascular compartment, replacing the interstitial compartment which is not needed due to its likely non-limiting role. Physiological data to parameterise the interstitial compartment of the placenta are lacking. The foetal vascular compartment in the placenta is connected to the foetal tissues accounted by 4 additional instances representing the foetal blood (arterial and venous), foetal lumped body compartment, and a foetal lumped lymph node compartment. This requires adding more “Index Values” to the “organ” index to represent the newly added organs. This in turn requires that values to be defined for the respective organs in all organ-specific parameters.

**Figure 14:**
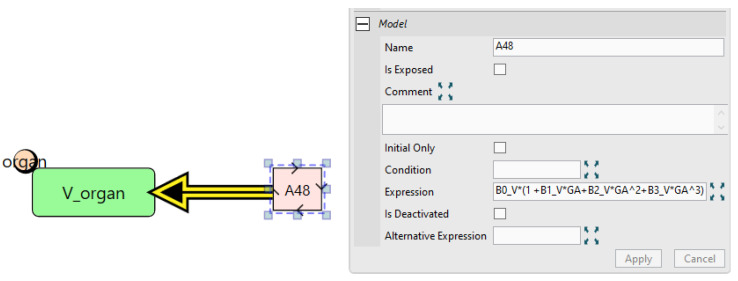
Repeated assignment of the organ volume “V_organ” parameter. GA: gestational age, B0_V, B1_V, B2_V, and B3_V are empiric constants. Those constants are fixed to zero for non-GA-dependent organs and were estimated for GA-dependent organs.

The next key adaptation that needs to be made is to incorporate gestational age (GA) dependent physiological changes. These changes include maternal adipose tissue and plasma volumes, foetal organ volumes and blood flow rates, maternal IgG synthesis rate, and placental endothelial FcRn levels. Time-varying physiological parameters can be implemented in Simcyp Designer through repeated assignment nodes which allow the parameter value to be updated at every solver time step based on GA as shown in Figure 14.

**Figure 15:**
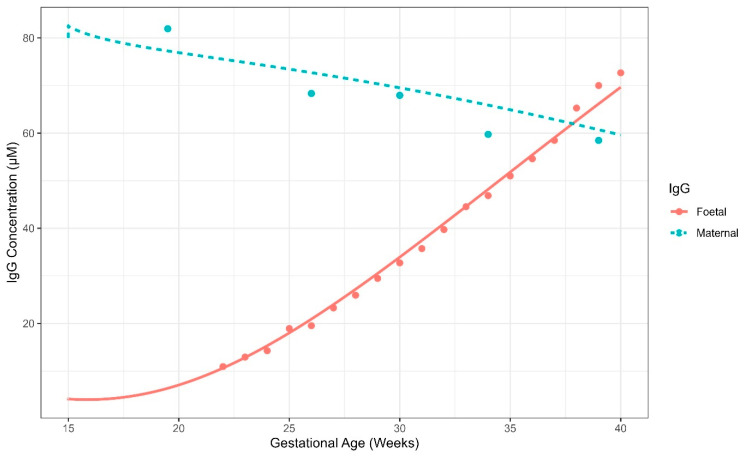
Simulated (lines) and observed (dots) IgG concentrations (µM) in the maternal and foetal blood from the gestational age of 15 weeks to delivery.

Some of the model parameters that were not available in literature and cannot be fixed to a reasonably assumed value were estimated using Simcyp Designer’s built-in parameter estimation engine based on available maternal and foetal IgG blood concentration data at various GAs [36] [37]. The final model was able to capture the data well as shown in Figure 15.

This case study demonstrates the utility of the reusable subgraphs for extending the model to include new organs as well as the repeated assignments for simulating dynamic physiological changes. Those two features allowed the original PBPK model to be readily adapted to incorporate pregnancy-specific compartments and parameters, with minor modifications. The final model allows the prediction of mAb PK in pregnant populations, despite data limitations.

### 5.3. Case Study 3: A Targeted Oligonucleotides Model

Oligonucleotides represent a novel therapeutic modality that has gained significant interest in recent years. These molecules modulate the expression of target proteins by degrading or modifying their encoding mRNA. The two main classes of therapeutic oligonucleotides are anti-sense oligonucleotides (ASOs), composed of single-stranded DNA, and small interfering RNA (siRNA), consisting of double-stranded RNA [38]. Although their mechanisms of action differ, the physiological factors influencing their PK are largely conserved. A major challenge in the development of oligonucleotide therapeutics is achieving efficient intracellular delivery to target tissues, primarily due to their large size and hydrophilic nature [39]. A significant breakthrough in addressing this challenge has been the development of liver-targeted delivery systems that exploit the asialoglycoprotein receptor (ASGPR) by conjugating the oligonucleotide molecule with N-acetylgalactosamine (GalNAc), a high-affinity ligand for ASGPR [40,41]. However, extending this delivery strategy to other tissues remains challenging, largely due to the high cost and labour-intensive nature of experimental methods required to evaluate the tissue-targeting efficiency of novel receptor-ligand pairs. Mechanistic PBPK modelling offers a promising, cost-effective alternative to predict the tissue distribution of oligonucleotides and evaluate the efficiency of different targeting strategies. In this case study, we demonstrate how the mAb platform model can be rapidly adapted to build an oligonucleotide PBPK model, allowing for the prediction of the PK of various oligonucleotide modalities (unconjugated and GalNAc-conjugated ASOs and siRNAs) across multiple species (mice and rats).

Given the extensive similarities in the mechanisms underlying the PK of mAbs and oligonucleotides, it is more insightful to think about the key differences that necessitate model adaptations. Three critical differences distinguish oligonucleotides from mAbs: (i) they exhibit significant plasma protein binding, profoundly influencing their tissue distribution and renal elimination, (ii) oligonucleotides do not bind to FcRn in endothelial cells, and (iii) their site of action is predominantly intracellular, making cellular uptake via specific and non-specific mechanisms a key determinant of pharmacological effect. These differences require three adaptation steps to the platform mAb model, as outlined below.

1. Modelling Plasma Protein Binding

A major adaptation involves incorporating plasma protein binding, as it significantly impacts the PK of oligonucleotides. Unlike mAbs, oligonucleotides bind extensively to plasma proteins such as albumin, reducing their free fraction and influencing tissue distribution and renal filtration. To capture this behaviour, we modified the model to separately account for the bound and unbound fractions of the drug.

To implement this, we introduced a new index, “pb”, with two values: “bound” and “free”. This index allows `model parameters to differ between the bound and unbound drug fractions. The most critical parameter is the MW (“MWkDa”), as it influences the calculation of two-pore parameters as well as renal filtration parameters. Therefore, the “pb” index was attached to these parameters, ensuring separate calculations for the bound and unbound fractions. Additionally, a new parameter representing the unbound fraction of the oligonucleotide in plasma (“fu_p”) was introduced. This was used to calculate the unbound fraction in the interstitial compartment assuming albumin is the primary binding protein.

**Figure 16:**
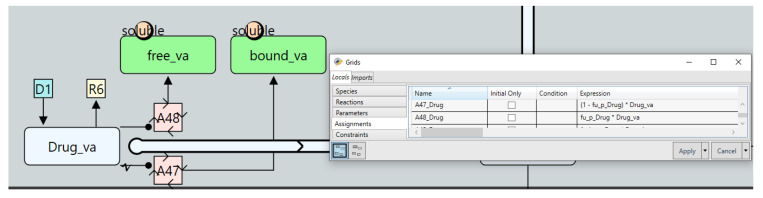
The use of repeated assignment nodes to compute time varying free and bound drug concentrations.

Finally, we need to calculate the free and bound concentrations of the drug (time-varying quantities) in both the vascular and interstitial compartment and use these to update the “twoPore” reaction governing the extravasation rate. Since these changes must apply to all tissues, the best place to implement them is the “Generic_Tissue” definition, as changes made here will automatically apply to all tissues. We first create two parameters in the vascular compartment; “free_va” and “bound_va” representing the free and bound drug concentration, respectively. Then, repeated assignments are used to calculate those quantities at each solver time step as shown in Figure 16. The same process is then applied to the interstitial compartment after which the “twoPore” reaction expression is updated to calculate the extravasation rate as the sum of the rates of the free and bound drug.

2. Disabling the FcRn recycling pathway

Oligonucleotides lack an FcRn binding domain, rendering the FcRn recycling pathway unnecessary in their pharmacokinetic models. To exclude this pathway, one can either remove the FcRn-specific reactions from the endothelial compartment or assigning a value of zero the FcRn association rate constant (*Kon_FcRn_*). The latter adjustment can be made directly within the workspace or via a virtual population (VPop) file.

3. Modelling intracellular uptake

**Figure 17:**
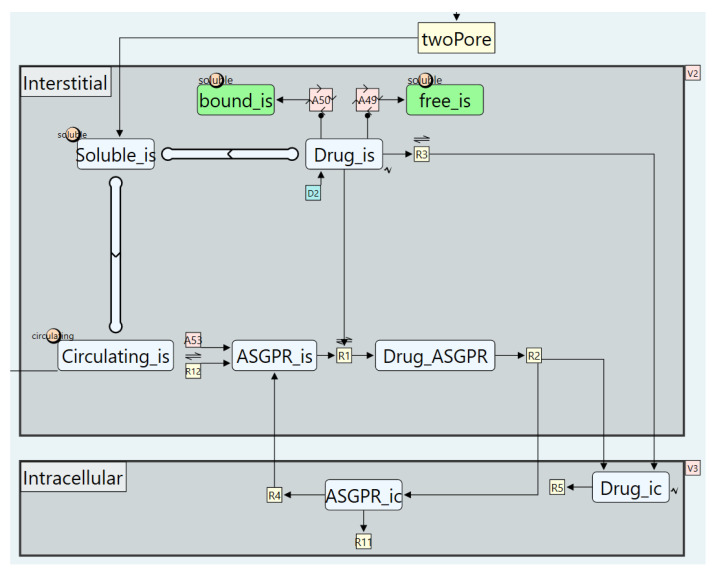
Cellular uptake and recycling of oligonucleotides as constructed within the “Generic_Tissue” subgraph definition.

Oligonucleotides undergo cellular uptake via two distinct pathways. The first is non-specific, non-saturable uptake through macropinocytosis, which occurs across all tissues and for all oligonucleotide modalities. This process is incorporated into the model as a first-order uptake process (see Figure 17). The second pathway is RME, which is specific to conjugated oligonucleotides and occurs only in tissues that express the corresponding receptor. Since the RME model is implemented within the “Generic_Tissue” definition, it applies to all tissues by default. However, receptor expression can be restricted to specific tissues by setting receptor abundance to zero in non-expressing tissues, effectively eliminating this uptake mechanism where it is not relevant. Similarly, unconjugated oligonucleotides can be simulated using the same model by setting the receptor binding parameter to zero.

4. VPop simulations

**Figure 18:**
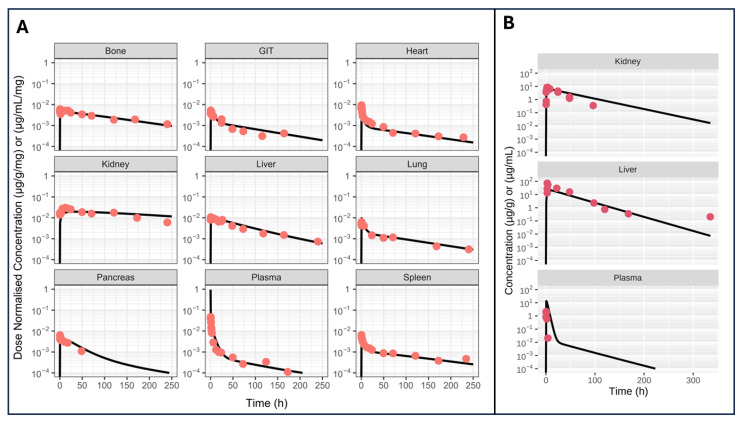
Model predicted (lines) and observed oligonucleotides concentration in various organs. Orange dot represent data from a non-conjugated ASO study in rats (**A**) while red dots represent data from a GalNAc conjugated siRNA in mice (**B**).

In this case study, we used VPop simulation features to simulate two oligonucleotides preclinical PK studies. First was a non-conjugated ASO study in rats [42], the second is a GalNAc conjugated siRNA in mice [43]. Model simulations versus observations are shown in Figure 18.

## 6. Discussion and Conclusions

This tutorial demonstrated the development of a generic biologics PBPK platform model and how it can be efficiently adapted to describe the PK of diverse biologics modalities and modelling scenarios. By leveraging a shared whole-body structure and a modular organ-level design, the platform minimizes redundancy in model construction while allowing for seamless customization. The case studies presented illustrate the model’s flexibility and broad applicability.

A key advantage of this platform is that it substantially reduces the time and effort required to construct mechanistic PBPK models for new biologics. Instead of building a new model from scratch, users can quickly modify relevant physiological and drug-specific parameters or introduce new sub-models, allowing rapid development of complex models while eliminating the need for error-prone manual adjustments. This efficiency is especially valuable in early-stage drug development, where the ability to quickly explore different tissue-targeting strategies or animal species extrapolations can significantly accelerate decision-making.

While the case studies illustrated in this tutorial focused on mAbs and oligonucleotides, the platform’s scope extends far beyond these examples. It can be readily adapted to other large molecules such as bispecific antibodies, antibody-drug conjugates, fusion proteins, or cell and gene therapies. Additionally, the modular design allows users to expand the platform by incorporating new mechanisms of action, target-binding kinetics, or tissue-specific physiological features without compromising the model’s core structure. The platform allows the seamless integration of intracellular dynamics or systems pharmacology models to explore therapeutic efficacy and safety.

Although this tutorial centres on the modular design and automation of biologics PBPK model assembly within the Simcyp Designer platform, we recognize that accurate parameterization is a critical step in model development, particularly for large molecules. The platform includes a parameter estimation engine that supports multiple algorithms [9], but detailed estimation workflows are beyond the scope of this tutorial. While some case studies involved parameter estimation, full details of the estimation strategy, including algorithm choice, fitting procedure, and sensitivity analyses, are provided in a separate manuscript currently under peer review. Together, these efforts demonstrate how model assembly and parameter refinement can be decoupled yet remain complementary within a transparent and reproducible PBPK modelling workflow.

Recent regulatory developments, notably the U.S. Food and Drug Administration’s (FDA) announcement to phase out mandatory animal testing for mAbs and other drugs, underscore a significant shift toward human-relevant drug development methodologies. This initiative aims to enhance drug safety, reduce development costs, and expedite the evaluation process. The FDA encourages the use of advanced computer simulations and artificial intelligence (AI) to predict a drug’s behaviour. As stated in the agency’s announcement: “Software models could simulate how a monoclonal antibody distributes through the human body and reliably predict side effects based on this distribution as well as the drug’s molecular composition” [44].

In this context, PBPK modelling stands out as a pivotal tool for nonclinical-to-clinical translation. While this tutorial demonstrates the use of cross-species virtual populations (Vpop files) to simulate PK in preclinical species, this functionality supports broader learning and validation workflows rather than implying a dependence on animal data. The Simcyp Designer Biologics platform facilitates the development of human PBPK models grounded in mechanistic principles and in vitro data, enabling the construction of human-relevant models without extensive in vivo animal studies. As regulatory guidance evolves, PBPK modelling offers a robust framework for reducing reliance on animal data while ensuring scientific and translational rigor.

Simcyp Designer does not currently incorporate a semantic or ontological validation layer to automatically flag biologically implausible constructs, such as reactions between incompatible compartments. This decision aligns with the platform’s primary goal of offering users a flexible graphical model building language. However, this flexibility places the responsibility on users to ensure the biological plausibility of their models through careful construction and validation. Moving forward, we are aware of the need for enhanced automation in model verification. The integration of AI-based validation tools is currently underway. These enhancements will be particularly valuable for increasing the usability and reliability of the tool in complex, large-scale models, as well as ensuring a more seamless transition from model building to simulation.

In summary, the biologics PBPK platform presented here offers a powerful, flexible, and efficient approach for modelling the pharmacokinetics of biologics across diverse therapeutic modalities and species. By significantly reducing the need for repetitive model construction, it enables researchers to focus more on addressing scientific questions and optimizing therapeutic strategies rather than spending time on manual model assembly and debugging.

## Figures and Tables

**Table 1:** An example VPop file.

ID	LABEL	P_A	P_B	S1_A	S1_B	S2_A	S2_B
1	Set 1	0.647911	6.422175	76.44275	2.585046	15.34369	4.826582
2	Set 2	0.619675	11.27114	74.00611	8.810178	94.17874	6.750496
3	Set 3	0.447106	10.09819	49.51735	5.76495	59.79229	1.762077
4	Set 4	0.50173	5.224387	43.71762	5.4861	34.39141	8.304497

## Supplementary Materials

The following supporting information can be downloaded at https://www.mdpi.com/article/10.3390/pharmaceutics17050604/s1: Supplementary S1: Graphical Model Object Types in Simcyp Designer; Supplementary S2: Quantity Lists Verification Checks; Supplementary S3: Reproducibility and Access.
