# Supplementary material for: Accelerating Biologics PBPK Modelling with Automated Model Building: A Tutorial
Source: Pharmaceutics. 2025 May 2;17(5):604. doi: 10.3390/pharmaceutics17050604 (PMC12115112; doi:10.3390/pharmaceutics17050604)
Supplement: Supplementary file 1 [file pharmaceutics-17-00604-s001.zip › pharmaceutics-3586076-supplementary.pdf]

Supplementary S1: Graphical Model Object Types in Simcyp Designer

Type	Visual Appearance	Description
**Nodes**		
**Species**	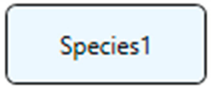	• Represent ODE states<br>• Initial State property is used to define its initial condition
**Parameter**	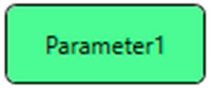	• Represent ODE parameters<br>• Has Value which can be set through initial or repeated assignments
**Reaction**	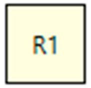	• Consume and produce Species<br>• Has Rate property which contain the rate expression
**Initial Assignment**	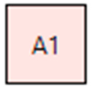	• Assigns values of evaluated expression to quantities (Species, Parameter) or Compartment once at the start of simulation.<br>• Can have a condition for conditional assignment
**Repeated Assignment**	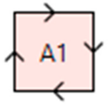	• Assign values of evaluated expression continuously at each solver time step.<br>• Can have a condition for conditional assignment
**Dosing Plan**	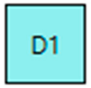	• Specifies times and amounts (or rates) of doses to Species.<br>• Can be reference other parameters in the model
**Index**	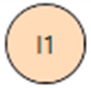	• Converts quantities into arrays when attached to them.
**Containers**		
**Compartment**	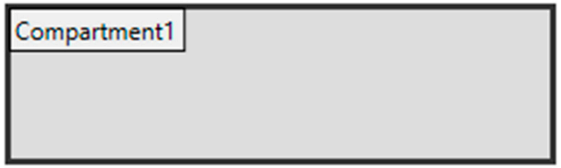	• Represent volume containing Species.<br>• Can be assigned initially or repeatedly

**Edges**		
**Substrate**	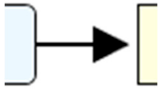	• Allows a Species to be consumed by a reaction<br>• Brings the quantity into scope for the reaction expression
**Product**	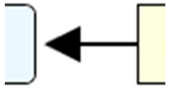	• Allow a Species to be produced by a reaction.<br>• Doesn't bring the Species into scope for the reaction expression.<br>• Can be used to connect an Assignment to a Parameter or a Compartment which allows them to be modified by the Assignment.<br>• Also connects dosing plans to Species indicating the Species is dosed by the dosing plan
**Growth**	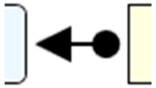	• Allows the quantity to be modified by the quantity modifying node<br>• Brings the quantity into scope for the quantity-modifying expression
**Modifier**	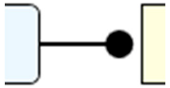	• Brings the quantity into scope for the quantity-modifying expression<br>• Does not allow the quantity to be modified by the quantity modifying node
**Inhibitor**	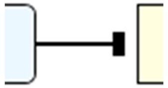	• Same as Modifier edge but with different visual appearance
**Quantity List Meta Edge**	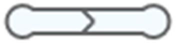	• Designates a quantity as a member of a quantity list, allowing it to inherit all of the list's quantity modifier actions.

Supplementary S2: Quantity Lists Verification Checks

Simcyp Designer enforces strict verification checks to ensure consistent indexing between quantity lists and their elements. These checks must pass before simulation execution or code generation can proceed. The key requirement for quantity lists is that each quantity list must be associated with exactly one index, referred to as the list index, which must be distinct from any indices assigned to the list's individual elements. Additionally, the range of the list index must match the total number of elements in the list.

For example, consider List1 as shown in Figure 6 of the manuscript. The list index in this case is "i", which is distinct because it is not shared by any of the list elements. "i" has Index Values of "A, B, C, D", giving it a range of 4, which matches the number of list elements. The order of these elements is explicitly defined through the Quantity Names property (see Figure S2.1). When elements of quantity lists such as List1 and List2 are correctly ordered (e.g., S1_A is the first element of List1 and S2_A is the first of List2), Simcyp Designer correctly maps corresponding components when unrolling reactions, e.g., generating a reaction from S1_A to S2_A, then S1_B to S2_B, and so on.

One additional index can be attached to one or more list elements. However, the range of the list index should still match the sum of the ranges of its elements. If additional indices are applied to the list elements, those indices must also be attached to all list elements and to the quantity list itself. This structured approach ensures that list-based modelling remains robust and reproducible, even when dynamically adapting list membership or model granularity.

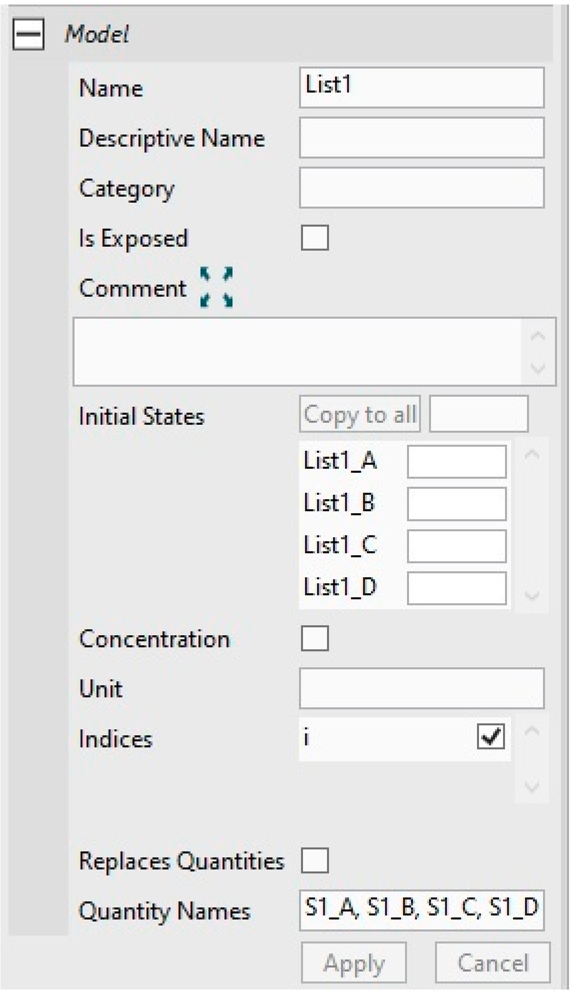

Figure S2.1: Quantity List Properties Panel in Simcyp Designer.

Supplementary S3: Reproducibility and Access

This tutorial was developed using the Simcyp Designer platform, a commercial software environment optimised for modular PBPK model construction. While the platform supports visual, code-free model assembly and promotes reuse through subgraph templates, its proprietary data structures and licensing conditions restrict direct public distribution of model templates and files.

Access via Evaluation License

To promote accessibility and reproducibility, the software provider, Certara, offers a limited-time evaluation license for interested researchers. This option enables users without an existing Simcyp license to explore the Designer environment and attempt to recreate the workflows described in this tutorial. Evaluation licenses can be requested by contacting the support team at: simcyp.support@certara.com

Availability of Model Templates

Researchers who already hold a Simcyp license and wish to reproduce the specific case studies described in this tutorial, including PBPK models for monoclonal antibodies, pregnancy, and oligonucleotide therapeutics, are invited to contact the corresponding author. Upon request, the model files and VPop parameter files can be shared (subject to licensing terms and usage rights).

These resources are designed to support independent reconstruction of the case studies using Simcyp Designer's visual modelling interface. Nonetheless, we note that the modelling approach described in this tutorial is intentionally built on modular, reusable components that correspond to discrete physiological or mechanistic elements (e.g., FcRn binding, target engagement, endosomal recycling). These modules are assembled visually in Simcyp Designer using well-defined subgraphs and parameter blocks that are explicitly described and illustrated in the tutorial figures. By outlining how these components are connected, users can reconstruct the model architecture in any capable software/language. This transparency in model logic and structure supports reproducibility and reuse across projects, even when access to proprietary files is limited.
